# Acknowledgement of Reviewers 2021

**DOI:** 10.3389/ijph.2022.1605135

**Published:** 2022-09-02

**Authors:** IJPH Editorial Office

**Affiliations:** Swiss School of Public Health (SSPH+), Zürich, Switzerland

The Editors of the International Journal of Public Health would like to thank all Reviewers in 2021 for the time they have spent and the valuable contributions they have made to ensure the scientific quality of the journal.

Abdishakur Abdulle, United Arab Emirates

Gboyega Abikoye, Nigeria

Karim Abu-Omar, Germany

Claudia Agnoli, Italy

Heba Al-Alwan, Switzerland

Aicha El Alaoui, Morocco

Kasim Allel, Switzerland

Lubna Alnasser, United States

Heresh Amini, Denmark

Doaa El Amrousy, Egypt

Jaithri Ananthapavan, Australia

Italo Angelillo, Italy

Daniela Anker, Switzerland

Fatme Al Anouti, United Arab Emirates

Songül Araç, Turkey

Seyed Mehdi Samimi Ardestani, Iran

Benedetta Armocida, Switzerland

Frank Baiden, United Kingdom

Luke Balcombe, Australia

Dinarte Ballester, Brazil

Mobolanle Balogun, Nigeria

Marco Bardus, Lebanon

Georg Bauer, Switzerland

Jorgen Bauwens, Switzerland

Laura Behan, United Kingdom

Hiram Beltrán-Sánchez, United States

Thomas Benz, Switzerland

Finaba Berete, Belgium

Machteld Wyss-Van Den Berg, Switzerland

Carina Berterö, Sweden

Christina Bethell, United States

Premananda Bharati, India

Marialaura Bonaccio, Italy

Alberto Borraccino, Italy

Florencia Borrescio-Higa, Chile

Achille Dadly Borvil, Canada

Elizabeth Bradley, United States

Cynthia Braga, Brazil

Damien Bricard, France

Denise Brookes, Australia

Heather Brown, Finland

Cecilia Calabrese, Italy

Mabel Carabali, Canada

Nicola Caranci, Italy

Massimo Finocchiaro Castro, Italy

Daniel Catalan-Matamoros, Spain

Serkan Catma, United States

Franco Cavallo, Italy

Mariachiara Di Cesare, United Kingdom

Daniel Chagas, Brazil

Michael Chaiton, Canada

Sanjiban Chakrabarty, India

Norshamliza Chamhuri, Malaysia

Angela Chang, China

Jean-Francois Chenot, Germany

Rajeev Cherukupalli, United States

Paolo Chiodini, Italy

Agnitra Roy Choudhury, United States

Prof Luke Clancy, Ireland

Natalie Colabianchi, United States

Paolo Contiero, Italy

Richard Cooper, United States

Alessandra Costanza, Switzerland

Florence Cousson-Gélie, France

Leopold Curfs, Netherlands

Astrid Czock, Switzerland

Sohel Daria, Bangladesh

Jean-Marie Degryse, Belgium

Jocelyn DeJong, Lebanon

Tom Deliens, Belgium

Matt DeLisi, United States

Daniel Demant, Australia

Michael Deml, Switzerland

Pablo Villalobos Dintrans, Chile

Julia Dratva, Switzerland

Sabine Drieskens, Belgium

Marie-Anne Durand, France

Anna Dzielska, Poland

Armstrong Dzomba, South Africa

Piet Van Eeuwijk, Switzerland

Sally El-Zahaby, Egypt

Jerome Endrass, German

Carla Enes, Brazil

Felix Ettensperger, Germany

Jean-Francois Etter, Switzerland

Mark Fabian, United Kingdom

Marta Fadda, Switzerland

Andrea Farnham, Switzerland

Omotayo Fatokun, Malaysia

Romulo Fernandes, Brazil

Pietro Ferrara, Italy

Antoine Flahault, Switzerland

Gilbert Fokou, Côte d’Ivoire

Geoffrey Fong, Canada

Maria Bonaventura Forleo, Italy

Annika Frahsa, Switzerland

Jane Frawley, Australia

Nancy Fullman, United States

Thierry Gagné, United Kingdom

Amandine Gagneux-Brunon, France

Jean Gajardo, Chile

Valentina Gallo, Netherlands

Behshid Garrusi, Iran

Teresa Gavaruzzi, Italy

Alan Geater, Thailand

Andrea Madarasova Geckova, Slovakia

Karin Geffert, Germany

Armin Gemperli, Switzerland

Francesco Gianfagna, Italy

Andrew Gibbs, South Africa

Stanton Glantz, United States

Juan Gómez-Salgado, Spain

Nathalia Gonzalez, Switzerland

Lara Goscé, United Kingdom

Amy Gower, United States

Rodrigo Goycolea, Chile

Igor Grabovac, Austria

Jakub Grabowski, Poland

Mathilde Gressier, United Kingdom

Aline Grünewald, Germany

Elodie Guillaume, France

Gabriel Gulis, Denmark

Ana Quiroga Gutierrez, Switzerland

Maria Isabel Gutierrez, Colombia

Jan Hattendorf, Switzerland

Margaret Haworth-Brockman, Canada

Alison Hayes, Australia

Ibrahim Haznedaroglu, Turkey

Carolyn Heckman, United States

Joachim Heinrich, Germany

Wanda Van Hemelrijck, Netherlands

Eilis Hennessy, Ireland

Johan Van Der Heyden, Belgium

Paula Hidalgo-Andrade, Ecuador

Heikki Hiilamo, Finland

Bjørn Evald Holstein, Denmark

Vincent Hooper, China

Rhea Ashley Hoskin, Canada

Cheng-Yang Hu, China

Dian Hu, United States

Zhiwen Hu, China

Daniela Husarova, Slovakia

Kristen Jafflin, Switzerland

Caitlin Jarrett, Switzerland

Anthony Jehn, Canada

Milos Jesenak, Slovakia

Søren Johansen, Denmark

Peter Jones, United Kingdom

Lisa Kakinami, Canada

Zarko Kalamov, Germany

Ramesh Kandimalla, India

Umit Kartoglu, Switzerland

Niranjan Kathe, United States

Warren Kealy-Bateman, Australia

Helen Keleher, Australia

Robin Van Kessel, Netherlands

Ali Talha Khalil, Pakistan

Olaf Von Dem Knesebeck, Germany

Peter Kolarčik, Slovakia

Marko Kolšek, Slovenia

Jaroslava Kopcakova, Slovakia

Michaela Kostičová, Slovakia

Petros Koutrakis, United States

Christian Krauth, Germany

Susi Kriemler, Switzerland

Vijay Krishnan, India

Alfgeir Logi Kristjansson, United States

Katarzyna Krot, Poland

Mark Kuczewski, United States

Nino Künzli, Switzerland

Victoria Leclercq, Belgium

Gobopamang Letamo, Botswana

Rosamund Lewis, Switzerland

Marta Lima-Serrano, Spain

Chien-Yu Lin, Taiwan

Christina Lindemann, Germany

Jutta Lindert, Germany

Marie Lindkvist, Sweden

Laura Llambi, Uruguay

Pablo Lopez, Argentina

Marian Loveday, South Africa

Daniel Lüdecke, Germany

Julia Ludwig, Germany

Macarlupu Jose Luis, Peru

Yuxi Luo, China

Nicole Lurie, Norway

Thenmozhi M, India

Shingai Machingaidze, Netherlands

Nicola Magnavita, Italy

Farnaz Mahdavian, Germany

Amyn Abdul Malik, United States

Francesca Mallamaci, Italy

Marco Ferdinando Martorana, Italy

Rainier Masa, United States

Joanna Masel, United States

Maria Masulli, Italy

Kirsten McCaffery, Australia

Laura McGuire, United Kingdom

Sally McManus, United Kingdom

Maria Jose Gonzalez Mendez, China

Sonja Merten, Switzerland

Doug Meyer, United States

Laetitia Minary, France

Pietro Amedeo Modesti, Italy

Ali Moghtaderi, United States

Ali Montazeri, Iran

Annie Montreuil, Canada

Matthis Morgenstern, Germany

Olaf Müller, Germany

Wudeneh Mulugeta, United States

Kirubel Mussie, Switzerland

Catherine Nagawa, United States

Mark Navin, United States

Nhung Nguyen, Vietnam

Kathryn Nicholson, Canada

Andrzej Nowakowski, Poland

Peter Odermatt, Switzerland

John O’Gorman, Australia

Isa Okajima, Japan

Claudio Olmos, Chile

Hiram Ortega, Mexico

Akihiro Otsuka, Japan

Ebenezer Owusu-Addo, Ghana

Secil Ozkan, Turkey

Raffaele Palladino, Italy

Salvatore Panico, Italy

Pallavi Pant, United States

Robbie Parks, United States

Farhana Parvin, India

Edgar Pazmiño, Ecuador

Sebastián Peña, Finland

Francisco Perales, Australia

Adjani Peralta, United States

Anastas Philalithis, Greece

Peter Von Philipsborn, Germany

Stefan Pichler, Switzerland

Carla Pires, Portugal

Cristina Bianca Pocol, Romania

Maria Prados, United States

James Prieger, United States

Parul Puri, India

Nouar Qutob, Palestine

Yael Rachamin, Switzerland

Siavash Radpour, United States

Sarah Rajkumar, Switzerland

Arkalgud Ramaprasad, United States

Jorge Ramirez, Chile

Neha Rathi, India

Ana Ribeiro, Brazil

Sandra Ribeiro, Portugal

Susan Rifkin, United States

Kirsti Riiser, Norway

Emmanuelle Robardet, France

Lesley Roberts, United Kingdom

Jose A. Rodas, Ecuador

Cristina Romero-Blanco, Spain

Suzie Roscoe, United Kingdom

Thomas Rosemann, Switzerland

Valentina Rotondi, Switzerland

Valentin Rousson, Switzerland

Miguel Ángel Royo-Bordonada, Spain

Andrew Rundle, United States

Jaesin Sa, United States

Pier Luigi Sacco, Italy

Carlotta Sacerdote, Italy

Anand Sahasranaman, United Kingdom

Jihene Sahli, Tunisia

Sherald Sanchez, Canada

Ahmed Sarki, Uganda

Gianluca Schiavo, Italy

Christian Schindler, Switzerland

Alexandra Schneider, Germany

Nina Schnyder, Switzerland

Jana Seblova, Czechia

Sean Semple, United Kingdom

Graham Serjeant, Jamaica

Miquel Serra-Burriel, Switzerland

Ruxandra Sfeatcu, Romania

Seyed Mahmood Taghavi Shahri, Denmark

Mansour Shamsipour, Iran

Akira Shibanuma, Japan

Wondimeneh Shiferaw, Ethiopia

Johannes Siegrist, Germany

Erik Sigmund, Czechia

Bruna Gonçalves C. Da Silva, Brazil

Michael Silverman, Canada

Vittorio Simeon, Italy

Jean Simos, Switzerland

Konstantinos E. Siomos, Greece

Vittorio Alessandro Sironi, Italy

Pierre Smith, Belgium

Denisa Sologon, Luxembourg

Anne Sordi, Brazil

Pedro Souza, Brazil

Giovanni Spitale, Switzerland

Susanne Stolzenburg, Germany

Saverio Stranges, Canada

Dagmar Strohmeier, Austria

Joseph Studer, Switzerland

Suzanne L. Suggs, Switzerland

Tintin Sukartini, Indonesia

Dewi Susanna, Indonesia

Boyd Swinburn, New Zealand

Viroj Tangcharoensathien, Thailand

Salina Torres, United States

Jordan Tustin, Canada

Vishnupriya V., India

Mohammad Valipour, United States

Beatriz Vallejo-Sánchez, Spain

Alessandro Vatrella, Italy

Gerry Veenstra, Canada

Justin Veuthey, Switzerland

Luisa Villa, Brazil

Nico Vonneilich, Germany

John Walsh, Thailand

Bethany Hipple Walters, Netherlands

Rajvi Jayant Wani, Canada

Jeremy Ward, France

Yukiko Washio, United States

Jingkai Wei, United States

Yaguang Wei, United States

Dean Whitehead, Australia

Ricky Wibowo, Indonesia

John Wilkerson, United States

Viktor von Wyl, Switzerland

Richard Brou Yapi, Côte d’Ivoire

Yusuf Yilmaz, Turkey

Apolinaras Zaborskis, Lithuania

Gerardo Zavala, United Kingdom

Yue Zhang, United States

Qingyang Zhu, United States

